# Sexual and reproductive health services use among adolescents in pastoralist settings, northeastern Ethiopia

**DOI:** 10.1186/s12913-023-09616-z

**Published:** 2023-06-22

**Authors:** Nejimu Biza Zepro, Nuruhussen Tahir Ali, Natalie Tarr, Araya Abrha Medhanyie, Daniel Henry Paris, Nicole Probst-Hensch, Sonja Merten

**Affiliations:** 1 Swiss Tropical and Public Health Institute, Kreuzstrasse 2, Allschwil, 4123 Switzerland; 2 University of Basel, Basel, Switzerland; 3 College of Health Sciences, Samara University, Afar, Ethiopia; 4 Afar Regional Health Bureau, Afar, Ethiopia; 5 Center for African Studies, University of Basel, Basel, Switzerland; 6 School of Public Health, College of Health Sciences, Mekelle University, Tigray, Ethiopia

**Keywords:** Adolescent, Sexual and reproductive health, Service use, Pastoralist, Afar, Ethiopia

## Abstract

**Background:**

Adolescents have special sexual and reproductive health (ASRH) needs and are susceptible to poor health outcomes. The global burden of ill sexual health includes a significant proportion of Adolescents. The existing ASRH services in Ethiopia and particularly in the Afar region are currently not well suited to meet the needs of pastoralist adolescents. This study assesses the level of ASRH service utilization among pastoralists in Afar regional state, Ethiopia.

**Method:**

A community based cross-sectional study was conducted from January to March 2021 in four randomly chosen pastoralist villages or *kebeles* of Afar, Ethiopia. A multistage cluster sampling procedure was used to select 766 volunteer adolescents aged 10–19. SRH services uptake was measured asking whether they had used any SRH service components during the last year. Data was collected through face-to-face interviews with a structured questionnaire; data entry was done with Epi info 3.5.1. Logistic regression analyses was used to assess associations with SRH service uptake. SPSS version 23 statistical software package was used for advanced logistic regression analyses to assess the associations between dependent and predictor variables.

**Results:**

The study revealed that two-thirds or 513 (67%) of the respondents are aware of ASRH services. However, only one-fourth (24.5%) of the enrolled adolescents used at least one ASRH service in the past twelve months. ASRH services utilization was significantly associated with gender (being female [AOR = 1.87 (CI 1.29–2.70)], being in school [AOR = 2.38(CI: 1.05–5.41), better family income [AOR = 10.92 (CI; 7.10–16.80)], prior discussions of ASRH issues [AOR = 4.53(CI: 2.52, 8.16)], prior sexual exposure [AOR = 4.75(CI: 1.35–16.70)], and being aware of ASRH services [AOR = 1.96 (CI: 1.02–3.822)]. Being pastoralist, religious and cultural restrictions, fear of it becoming known by parents, services not being available, income, and lack of knowledge were found to deter ASRH service uptake.

**Conclusion:**

Addressing ASRH needs of pastoralist adolescents is more urgent than ever, sexual health problems are increasing where these groups face broad hurdles to SRH service uptake. Although Ethiopian national policy has created an enabling environment for ASRH, multiple implementation issues require special attention to such neglected groups. “Gender-culture-context-appropriate” interventions are favorable to identify and meet the diverse needs of Afar pastoralist adolescents. Afar regional education bureau and concerned stakeholders need to improve adolescent education to overcome social barriers (e.g. humiliation, disgrace, and deterring gender norms) against ASRH services through community outreach programs. In addition, economic empowerment, peer education, adolescent counseling, and parent-youth communication will help address sensitive ASRH issues.

## Background

According to the World Health Organization (WHO), adolescents are young people between the age of 10 and 19 [1–3], which is a unique and critical period in an individual’s life. They account for 20% of the world’s population, 85% of whom live in developing countries. The time period of adolescence represents the transition between childhood and adulthood requiring special attention. It is characterized by significant physiological, psychological and social changes representing a vulnerable phase for adolescents, but it also offers opportunities affecting life prospects. The health needs of young people have been given a little attention in global public health policy because adolescents are considered healthy, without a heavy “burden of disease” [4–7]. However, 6% of the global burden of disease and injury is seen among adolescents and attributed to alcohol use (7% of DALYs), unsafe sex (4%), iron deficiency (3%), lack of contraception (2%), and illicit drug use (2%) [8]. Many adolescents die prematurely each year; an estimated 1.7 million lose their lives to accidents, violence, pregnancy-related complications, and other diseases [2].

Universal access to SRH services is a human right, central to realizing the 2030 Agenda, Sustainable Development Goals (SDGs) relating to good health, wellbeing, and gender equality [9]. Reproductive rights are human rights protected by national and international laws and human rights documents [10]. Thus, all groups must be granted access to SRH services, so they are free to make informed choices and decisions [11–13]. Adolescent sexual and reproductive health (ASRH) problems comprise a major component of the global burden of ill sexual health [14]. Yet, ASRH is increasingly neglected despite its global health importance [3, 15].

The first sexual intercourse or “sexual debut” is considered a stepping-stone to later intimate relationships and transition to adulthood [16]. According to current US national conventions, a debut is regarded as “early” if it occurs before the age of 15, “normative” if it occurs between the ages of 15 and 19, and “late” if it occurs beyond the age of 19 [17]. Studies provide consistent evidence that adolescents with an early debut of sexual experimentation and without adequate knowledge and access to SRH services are less likely to use contraception. This, in turn, is associated with problematic outcomes like having several sexual partners, engaging in unprotected sex, resulting in unplanned pregnancy, sexually transmitted illness (STIs), and development of depression [18, 19]. So, adolescents have long-term SRH needs and expectations. Developing ASRH support requires the establishment of a solid foundation for long-term adolescent health and wellbeing [20], 16]. Unmet SRH needs in adolescence are linked to poor health outcomes like unintended pregnancy and bring long-lasting impact on the educational, economic and social wellbeing of Adolescents [17, 18].

Studies in sub-Saharan Africa show that adolescent women account for up to 60% of hospitalizations for abortion complications and newly acquired HIV infections [21, 22]. Adolescent (15 and 19) births are over 12.8 million and majority of these pregnancies are unplanned[23]. More than 2 million adolescents undergo unsafe abortions each year [24–26]. Moreover, Ethiopia is also one of the countries with the highest adolescent early marriage. A study conducted in Ethiopia exposed that 17% of respondents had married before age 15 and 30% had married at ages of 15–17 [27]. Adolescent motherhood is prevalent in Ethiopia, primarily due to high rates of child marriage. According to the most recent Ethiopian Demographic and Health Survey (EDHS), in 2016, 13% of adolescent girls aged 17, 20% of girls aged 18, and 28% of girls aged 19 were already mothers or pregnant [28]. Ethiopian adolescents aged 15–19 years had a total fertility rate of 63 births per 1000, which is substantially higher than the global average of 46 [28, 29]. The median age of sexual debut for nomadic youth in Kenya and Ethiopia is as low as 15 years [19].

Ethiopian policy has created an enabling environment for improving adolescent reproductive health services, but implementation is not widespread in all regions [1]. Adolescents in Ethiopia are exposed to a variety of SRH problems such as lack of adequate knowledge, lack of open discussion about sexual issues, low decision-making power of young girls, bad cultural norms, poor logistics, insufficient funding for primary health care, unprotected premarital sex, early marriage, early pregnancy, unsafe abortions, STIs, HIV/AIDS, drug abuse, and crime [29–32]. The leading causes of death in adolescents are complications due to unsafe abortions, substance abuse, mental health problems, insufficient/poor quality nutrition, violence, and accidents. HIV and maternal mortality are the two most important ASRH problems for adolescents in developing countries [33, 34]. Sexual gender based violence and commercial sex work have also become a common phenomenon [35].

There is some evidence suggesting socio-cultural context in pastoral settings plays an important role in the use of SRH services. This is because pastoral settings often have certain cultural practices, beliefs and norms that can influence perceptions of health and illness and willingness to use health services. Little is known about the effectiveness of interventions aimed at improving SRH service use in pastoral settings, particularly among adolescents. However, the evidence on the relationship between socio-cultural contexts and the use of health services by young people and adolescents in pastoral settings is dearth[36]. This is likely due to the diversity of pastoral communities and the lack of research on this topic [37].

The pastoralist Afar region scores lower on SRH health indicators in comparison to agrarian communities of Ethiopia. The prevalence of female genital cutting/mutilation (FGC/M) is 98.4%, with subsequent lifelong complications during childbirth, pain during sexual intercourse, excessive bleeding, infection, and urine incontinence. The median age at marriage is 16.8 years and 23% of those married teenagers give birth as a teenager; both figures are the highest in Ethiopia. Early marriage is associated with stress leading to higher rates of postpartum depression, resulting in higher rates of suicide, low educational achievement, medical complications, subsequent infertility, low labor force participation, reduced earnings, violence, and marital failure. Meanwhile, the prevalence rate of using contraceptives lies at only 11.6% [28].

Ethiopia is committed to meeting the SRH needs of the country’s largely neglected Adolescent population [29, 38]. ASRH needs of pastoralist communities are not well understood or documented due to a lack of adequate research findings. In addition, evidence of low and inappropriate use of ASRH services by pastoralist communities is also not well known. Therefore, adolescents and youths (AYs) in pastoralist settings could potentially benefit from key findings of a study by having their voices and experiences heard, which can empower them to demand better ASRH services and to make informed decisions, design contextual ASRH programs, and develop ASRH services that are culturally appropriate and accessible in remote settings. Understanding the barriers and facilitators can help identify areas for improvement and guide the development of targeted interventions for better utilization of ASRH service uptake. These findings are vital in designing, implementing, and monitoring culture-sensitive, adolescent-friendly services.

## Methods

### Study area

Afar national regional state is located in northeastern Ethiopia, bordering Djibouti to the east and Eritrea to the north. The region has an arid and semi-arid environment with low and erratic rainfall; the average annual rainfall in the semi-arid zones is less than 500 mm and 150 mm in the arid zones. Afar is increasingly affected by drought. The production system of the Afar region is dominated by pastoralism (90%) and agro-pastoralism (10%).[39]. The region has almost 2 million inhabitants of which 44% are females [40]. Afaraf is a widely spoken local language. Food insecurity affects a large majority of the population, and illiteracy is widespread. Poor health-care coverage, limited access to paved roads, and shortage of drinking water are widely reported. Maternal and adolescent health services are inaccessible due to geography, security concerns, linguistic hurdles, low health care acceptance, and cultural barriers of pastoralist community members [41–44]. The study was carried out in Mille district, Ethiopia. Mille district is located along the main road to Djibouti and is around 550 km from the capital city Addis Ababa and 65 km from the Afar regional capital Samara. With an area of 5,345.71 square kilometers, Mille has a population density of 16.96. The total population of the district is estimated to be 113,912, of which 38,730 are adolescents according to 2020 Central Statistical Agency (CSA) population projection. The district has 12 *kebeles*^2^ (2 urban and 10 rural). There is one maternity hospital, four health centers, ten health posts, five private clinics, and numerous independent drug vendors. ASRH services are available in most of these health institutions. However, specially trained staff offering SRH services to married and unmarried adolescents and young people is barely available.

### Study design

A community-based, cross-sectional, quantitative study design was used to assess associations with ASRH service use in Mille district. The study was carried out from January to March 2021. All participants provided written informed consent and eligible adolescents (boys and girls ages 10–19 years) residing in the Mille district for more than six months represented the source population.

### Sample size determination

Sample size estimations were conducted to estimate prevalence with an anticipated precision considering proportion (p) for ASRH services uptake and associated factors among adolescents to be 39.3% from a study conducted in Ancahr district, eastern Ethiopia [45]. The required sample size for the study was determined by using the single population proportion formula: n = Z^2^pq/d^2^. A 5% margin of error, 95% confidence level, design effect of 2, and a non-response rate of 10% was assumed to calculate the final sample size of 766.

### Sampling procedure

A multi-stage cluster sampling technique was employed to proportionally recruit adolescents from the whole district. A health extension worker in the community helped us to obtain a list of all households for each kebele. This list served as a sampling frame from which the number of households was randomly selected. In the first stage, 4 out of 12 kebeles in the district were selected randomly. In the second stage, households in each selected kebele were selected randomly by the lottery method. Adolescents (10–19 years) present in the household at the time of the visit that consented to participate were interviewed. If there is, more than one eligible adolescent in a household, one was selected by lottery. Interviewers revisited eligible study participants when they were away from home during recruitment. If the interviewers failed to meet the adolescents, the household was documented and replaced by the next household in a clockwise direction. This sampling technique was appropriate to reach pastoralist adolescents who are widely scattered.

### Data collection tool and procedures

A standardized structured questionnaire was developed by collecting questions from various relevant sources and adopting them after customizing them to the study context [21, 22, 25, 33, 46, 47]. Questions included socio-demographic and other individual characteristics potentially associated with ASRH service utilization. ASRH service utilization was measured with the following question: *Have you ever used any sexual reproductive services in the last 12 months? (Yes/No).* The types of services used further validated positive “yes” response. These included information and counseling, contraceptive use, voluntary HIV testing and counseling, care for abortion, and STI testing and treatment. The data was collected though face-to-face interviews with the structured survey tool. The questionnaire was developed in English first, translated into the local language *Afaraf* by a qualified language expert who is familiar with the subject matter and then back translated into English to ensure consistency. The language translations were performed in consultation with the PI (NTA) in order not to change the contextual meaning.

### Operational definitions

**ASRH attitude**: Positive or favorable: when the adolescent met or exceeded the mean score (66.48) of 24 attitude questions measured with a Likert scale (1–5). All questions were pilot tested, and possible contextual adjustments were made.

#### ASRH knowledge

Knowledge of SRH service use was assessed by asking adolescents whether they were aware of specific SRH services. A summative score with eight items SRH service indicators was used to assess the overall knowledge. The knowledge ranged from one (minimum) to eight (maximum).

#### ASRH service awareness

Adolescents who were aware of the availability of one or more SRH services indicated that they were aware of SRH services.

#### Sexual activity

means having had penetrating heterosexual relations within the previous 12 months.

#### ASRH services use

Measured by the dichotomous response (yes/no) among adolescents in the past twelve months. ASRH services includes family planning, sexually transmitted illness (STI) and treatment of SRH related problems, voluntary counselling and testing, antenatal care, delivery care, post-natal care, abortion care, post-abortion care, and condom use.

#### Average income

Livestock herding remains the most important source of income for people in pastoral settlements and is not easy to quantify in birr. The income was estimated by taking the number of livestock they own into account.

### Data collection and quality control

The questionnaire pretest and piloting was applied to 39 adolescents (5% of the sample), who came from similar backgrounds in the nearby Ada’ar district; few modifications such as approach to the questions, skip pattern, and sequence adjustment were made. Eight diploma and two Bachelor nurses who know the culture and the language were recruited for data collection and supervision, respectively. The principal investigator trained the data collection team on the tool handling and the scientific procedures for two days. Communication between the Principal Investigator (PI) and the data collecting team was in *Afaraf*. The PI (NTA) did supervision and checking of the filled-in questionnaires on daily basis. Finally, each questionnaire collected in the field was checked for completeness, missing values, and unlikely responses.

### Data processing and analysis

All returned questionnaires were checked for completeness and consistency manually. Thereafter, the field paper-based data was entered into a computer database using EPI INFO version 3.5.1 and the cleaned data was exported to SPSS version 23 computer software package for advanced analysis (IBM Corporation, Armonk, NY, USA). The descriptive statistics was presented in the form of tables and figures. Binary logistic regression analysis was performed to evaluate the crude association between dependent and independent variables. The main assumptions of logistic regression analysis were checked, e.g. the binary nature of the outcome variables. The outcome variable (ASRH service use) should be dichotomous with only two categories (yes or no), 1 or 0. We belive that we estimated adequate sample size to ensure reliable estimates and standard errors. We checked presence of outliers through plots that unduly influence the results of the analysis. Independence of observations: Each observation should be independent of other observations in the data set. The fit of the model was tested using goodness-of-fit tests or by comparing the observed data with the values predicted from the model. Absence of multicollinearity: The predictor variables should not be highly correlated with each other. The variance inflation factor (VIF) was used to diagnose collinearity.

Finally, the variables that showed an association in the binary logistic regression analysis or whose p-value was less than or equal to 0.25 were included in a multivariable logistic regression model to identify significant associations and confounding factors with the dependent outcome. Adjusted odds ratio and 95% confidence intervals are reported and *p* values less than or equal to 0.05 in multivariable logistic regression analysis were declared as statistically significant associations.

## Results

### Socio-demographic and socio-economic characteristics of the study population

Seven hundred and sixty-six adolescents participated in the study making 100% response rate, 442 (57.7%) were males and 324 (42.3) were females. The mean age of the adolescents was 15.45 ± 1.89 years. The Afar ethnic group was represented by 614 (80.2%) of the adolescents and 709 (92.6%) were Muslim. The majority of the adolescents, 420 (59%) boys and 288 (41%) girls, were single. More than four-fifths, 659 (81.1%), had completed formal education. Six hundred and seventy-seven (88.4%) adolescents were living with both parents and about two-thirds or 477 (62.3%) of respondents’ an average family income was less than 1205 ETB a month (Table 1).

**Table 1:** Socio-demographic characteristics of male and female adolescents, Afar, northeast Ethiopia, 2021

Variable	Gender		P value of Pearson chi2*
	Male n (%)	Female n (%)	
**Age category**			< 0.001
10–14 years	171 (39)	123 (38)	
15–19 years	271 (61)	201 (62)	
Mean	15.45 ± 1.89		
**Religion**			0.22
Muslim	405 (91.6)	304 (94)	
Christian	37 (8.4)	20 (6)	
**Ethnicity**			< 0.001
Afar	336 (76)	278 (86)	
Oromo	30 (6.8)	11 (3.4)	
Amhara	53 (12.5)	17 (5.2)	
Others	23 (5.05)	18 (5.5)	
**Marital status**			0.01
Single	420 (95)	288 (89)	
Married	20 (4)	29 (9)	
Others	2 (1)	7 (1.6)	
**Schooling status**			0.27
Student	375 (85)	284 (88)	
Out of school	67 (15)	40 (12)	
**Educational status**			< 0.001
No formal education at all	67 (15)	40 (12)	
Primary education (1–8)	253 (57)	260 (80)	
Secondary education (9–12) & above	122 (28)	24 (8)	
**Mothers’ educational status**			0.25
No formal education	302 (69)	226 (71)	
Primary education (1–8)	98 (22)	78 (24)	
Secondary education (9–12)	24 (6)	10 (3)	
Above secondary education	13 (3)	5 (2)	
**Guardianship**			0.08
parents	383 (87)	294 (91)	
others	59 (13)	30 (9)	
**Family income (per month)**			0.57
< 1205 ETB	279 (63)	198 (61)	
≥1205 ETB	163 (37)	126 (39)	

*Fisher’s exact P-value was calculated for cells with small sample sizes

### Adolescent sexual and reproductive health related information and practice

Approximately two-thirds, 524/766 (68.4%), of the respondents had previously heard about ASRH services and radio was mentioned to be the dominant source of information. Out of this number, 442 (58%) and 324 (42%) were boys and girls respectively. Four hundred and fifty-eight, 458/766 (59.8%), were found to be sexually active during the last 12 months. Two thirds of the adolescents 517 (67.5%) had never discussed ASRH topics with their peers, health worker, or parents. Among this subgroup, 324/517 (63%) were males. Regarding their previous sexual practices, 279 (63%) boys and 179 (55%) girls had had sexual intercourse and only 70 (21.6%) of girls used condoms during sexual intercourse. About one-third or 249 (32.5%) and 385 (50.3%) of the respondents had talked about ASRH concerns with their families or peers, respectively (Table 2).

**Table 2:** ASRH by socio-demographic and SRH characteristics of pastoralist adolescents stratified by gender, Afar, Ethiopia, 2021.

	Male			Female		
	ASRH service use			ASRH service use		
	**Yes, N (%)**	**No, N (%)**	P-value	**Yes, N(%)**	**No, N (%)**	P-value
***Total***	**91(21)**	**351(79)**		**97(30)**	**227(70)**	
***Marital status***			< 0.001			< 0.001
Never married	78 (19)	342 (81)		72 (25)	216 (75)	
*Ever married*	13 (59)	9 (41)		25 (69)	11 (31)	
**Schooling status**			0.011			< 0.001
Student	85 (23)	290 (77)		95 (33)	189 (67)	
Out-of-school	6 (9)	61 (91)		2 (5)	38 (95)	
**Educational status**			< 0.001			0.001
No formal education at all	6 (9)	61 (91)		2 (5)	38 (95)	
Primary education	40 (16)	213 (84)		89 (34)	171 (66)	
Secondary education & above	45 (37)	77 (63)		6 (25)	18 (75)	
**Guardianship**			< 0.001			0.012
Parents	62 (16)	321 (84)		82 (28)	212 (72)	
Others	29 (49)	30 (51)		15 (50)	15 (50)	
**Sexual exposure**			< 0.001			< 0.001
Ever had sex	85 (26)	239 (74)		77 (39)	123 (62)	
Never had sex	6 (5)	112 (95)		20 (16)	104 (84)	
**Media exposure to SRH**			< 0.001			< 0.001
Ever exposed	89 (34)	176 (66)		92 (36)	167 (64)	
Never exposed	2 (1)	175 (99)		5 (8)	60 (92)	
**Perceived risk for acquiring HIV**			< 0.001			< 0.001
high	45 (52)	42 (48)		23 (61)	15 (39)	
low	42 (27)	114 (73)		59 (28)	154 (72)	
**Condom use**			0.606			0.018
Yes	22 (22)	76 (78)		29 (41)	41 (59)	
No	69 (20)	275 (80)		68 (27)	186 (73)	
**ASRH discussion**			0.000			< 0.001
Yes	83 (70)	35 (30)		68 (52)	63 (48)	
No	8 (2)	316 (98)		29 (15)	164 (85)	

Ever married is used here to indicate Adolescents who have been married at least once in their lives although their current marital status may not be “married” (divorced, widowed, separated…)

### Adolescent sexual and reproductive health service uptake

**Fig. 1:**
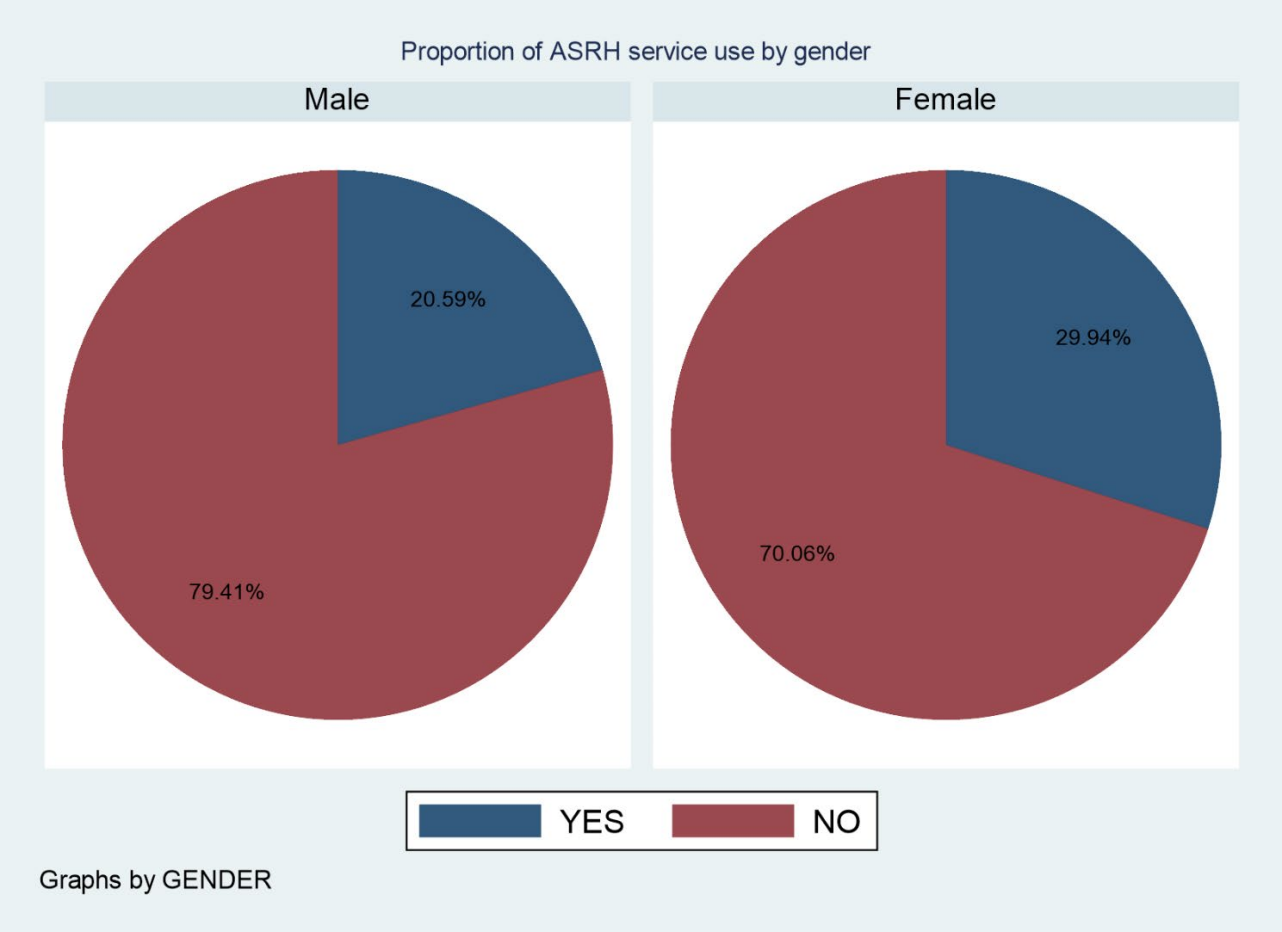
Pie chart showing proportion of pastoralist adolescents who ever used ASRH services in Afar, Ethiopia

**Fig. 2:**
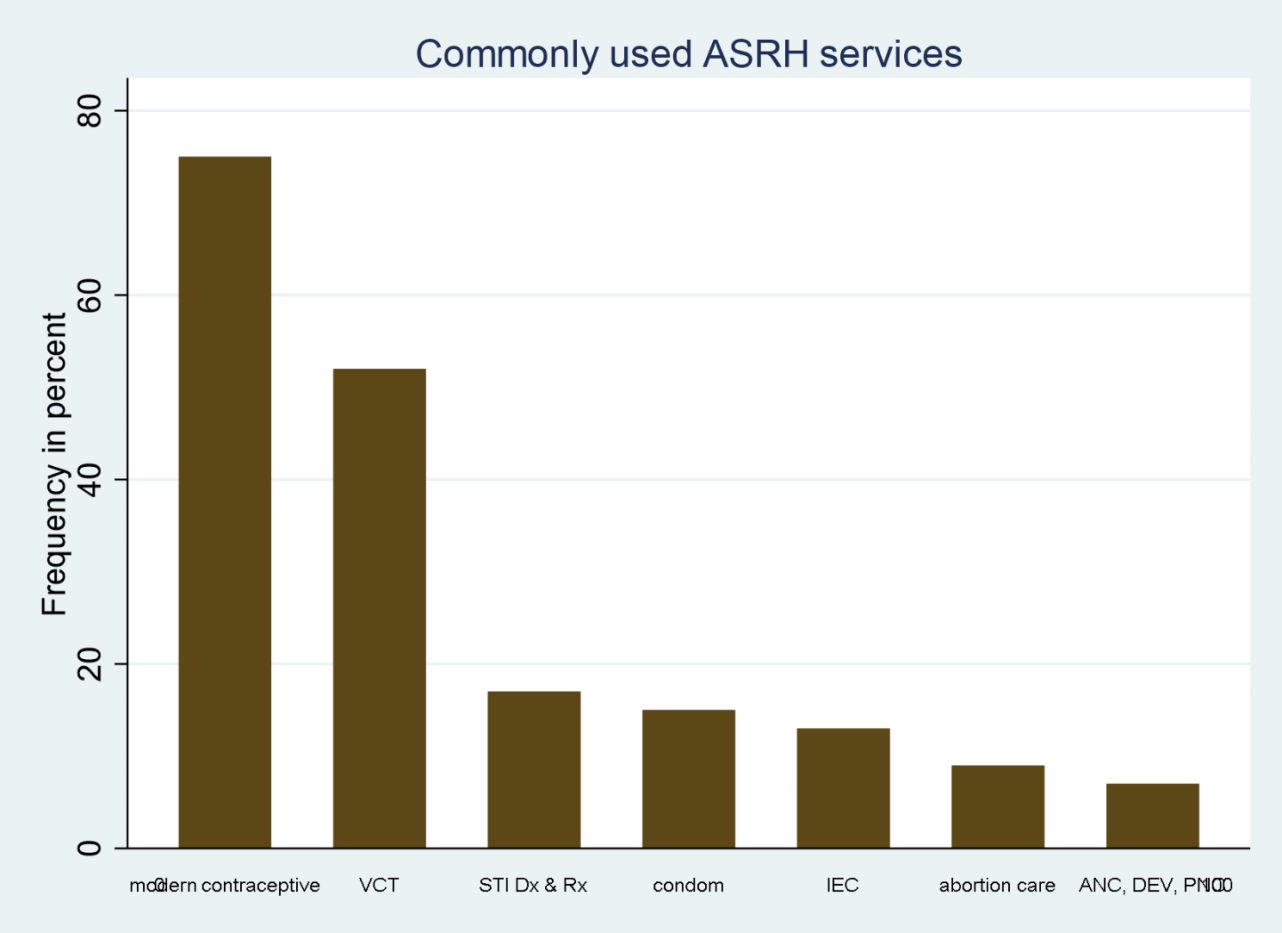
Bar graph showing list of commonly used SRH services among pastoralist adolescents in Afar, Ethiopia

Out of all participants, only 188/766 (24.5%) (95%CI: 21.5–27.8%) used ASRH services within the last twelve months. Adolescent girls were more likely to use ASRH services, namely 97 (30%) in comparison to 91 (21%) boys (Fig. 1 below). Family planning, Voluntary Counseling and Testing (VCT), STI diagnosis/treatment, abortion care, and general counselling services were used by 39.9%, 27.7%, 9.4%, 4.8%, and 6.9% of Afar adolescents, respectively (Fig. 2). Among the modern contraceptive service users, 34 (37.7%) used emergency pills, 21 (23.4%) used an injectable, 17 (11%) used oral pills (COC), 15 (16.7%) used condoms, 3 (3.3%) used an implant, and 11 (12.2%) used dual methods. The main barriers that refrained adolescents from accessing SRH services were: unawareness (“never thought of the service”) 152 (26.3%); religion/cultural restrictions 112 (19.4%); stigma (“fear of families”) 88 (54%); need (“service not necessary”) 89 (15.4%); availability (“no ASRH service in the vicinity “) 80 (13.8); and lack of knowledge 57 (9.9%).

### Factors associated with ASRH services use

Sociodemographic and economic characteristics such as age, ethnicity, marital status, and families educational level were not statistically significantly with ASRH service utilization. However, gender, religion, school attendance, educational status, housing situation, and family income showed a statistically significant association with ASRH service utilization. However, gender, religion, being in school, highest educational attainment, living arrangements, and family income were found to be statistically significant with ASRH services use. Female adolescents had higher odds of using ASRH services than male counterparts [AOR = 1.87 (CI 1.29–2.70)]. Adolescents, who were currently enrolled in school, had 2.4 times higher odds of using ASRH services [AOR = 2.38(CI: 1.05–5.41) than those who are out of school. Adolescents from financially better-situated families were almost 10 times more likely to use ASRH services [AOR = 10.92(7.10–16.80)] compared to individuals who come from a relatively poor family.

About 85 (70%) of young men adolescents who engaged in prior discussions did use SRH services compared to only 52% of young women among those who participated in the discussions (see Table 3 below). Adolescents who had engaged in prior discussions on ASRH issues had 4.5 times higher odds of using ASRH services [AOR = 4.53(CI: 2.516, 8.16)] than those who had no experience of talking about ASRH related topics. Among sexually active adolescents, the odds of using SRH service was 4.75 times [AOR = 4.75(1.35–16.70)] higher than among those who had not been sexually active in the past twelve months. Those who were aware of ASRH [AOR = 1.96(CI: 1.0-3.82)] and had a high-risk perception for a HIV infection [AOR = 4.51(CI: 2.14–9.47)], were also found to have significant associations with ASRH service utilization. Pastoralist adolescents appeared to be influenced by their religious belief to not use ASRH services. Christians were nearly 13 times more likely to use ASRH services than Muslims (AOR, 12.93, 95% CI, 6.00-24.62).

**Table 3:** Bivariable and multivariable analysis of ASRH service utilization and associated factors among adolescents in Mille district, Afar, northeast Ethiopia, 2021

Variable	ASRH service use		COR (95% CI)	AOR (95%)
	**Yes**	**No**		
**Gender**				
Male	91	351	1	1
Female	97	227	1.65(1.18–2.29)*	1.87(1.29–2.70)**
**Religion**				
Muslim	145	564	1	1
Christian	43	14	11.95(6.362–22.44)*	12.93(6.00-24.62)**
**Currently enrolled in school**				
Yes	180	479	4.65(2.22–9.752)*	2.38(1.05–5.41)**
No	8	99	1	1
**Educational status**				
No formal education	8	99	1	1
Primary education	129	384	1.60(1.08–2.37)*	2.07(0.79–5.41)
Secondary education & above	51	95	6.64(3.00-14.74)*	2.56(1.12–5.88)**
**Current living arrangement**				
With both parents	144	533	1	1
Not with both parents	44	45	3.62(2.30–5.70)	2.65(1.43–4.88)
**Family Income per month**				
< 1205 ETB	38	439	1	1
≥1205 ETB	150	139	12.46(8.33–18.67)*	10.92(7.10–16.80)**
**Awareness towards ASRH**				
Yes	162	351	4.03(2.58–6.30)*	1.96(1.00-3.82)**
No	26	227		
**Ever had sexual intercourse**				
Yes	162	362	3.72(2.38–5.81)*	0.55(0.15–2.07)
No	26	216		
**Condom use**				
Yes	51	117	1.47(1.00-2.15)*	0.97(0.51–1.86)
No	137	461		
**ASRH discussion**				
Yes	151	98	19.99(13.13–0.43)*	4.53(2.52–8.16)**
No	37	480	1	1
**Sexual exposure in the past 12 months**				
Yes	158	300	4.88(3.20–7.45)*	4.75(1.35–16.70)**
No	30	278	1	1
**Perceived risk of acquiring HIV (n = 524)**				
Yes	57	57	3.52(2.38–5.35*)	4.51(2.15–9.47)**
No	101	298		
**ASRH Media exposure**				
Yes	181	343	17.72(8.18–38.38)*	13.28(1.35-130.86)**
No	7	235		

Note: *: significant variable bivariable, **significant at multivariable analysis

## Discussion

The study revealed that only one-fourth of the adolescents in Mille district used SRH services in the previous twelve months. Services utilization was significantly associated with being female, educated, having a better family income, prior knowledge on SRH issues, and pre-existing awareness about ASRH services. Religious and cultural restrictions, fear of families, unavailability of service, and lack of knowledge were major reasons that prevented SRH service uptake among pastoralist adolescents. The practitioner is frowned upon so fear of using ASRH services. Research findings from studies done in Nigeria and India show that the majority of adolescents who contracted STIs did not seek ASRH services for several reasons: Shame, embarrassment, stigma, high cost of services, negative provider attitudes, and perceived lack of confidentiality. Some adolescents feared being teased and faced discriminating behavior by health care providers [48]. In Kenya, cultural attitudes, family restrictions, shyness, and fear were all factors that influenced adolescent girls’ access to SRH services [49].

Our study prevalence of 24.5% accessing SRH services was consistent with previous studies from the Badewacho district, Hadiya zone (29%) [50], and the Machakel district, Gojam zone (21.5%) [51]. However, the Mille district prevalence of SRH services attendance was lower than those in Harar (63.8%) [52], Bahir Dar (32%) [53], and Adama town (34%) [54]. One likely explanation for this finding could be the differences in study populations and education-levels of the communities; the studies in Harar and Bahir Dar were conducted among high school adolescents in urban settings, while our study was community-based and includes both in-school and out of school adolescents in pastoralist rural villages. High school students in urban settings may have better access to education, thus better knowledge regarding ASRH issues. In addition, the places they can get the services at are likely to be more accessible compared to the places for the out of school pastoralist adolescents. These findings can be used to inform the development of targeted interventions aimed at increasing ASRH service use in both rural and urban settings of pastoralist communities.

The study revealed that females were more likely to utilize SRH services as compared to males. This could be related to a stronger risk perception towards an unplanned pregnancy that motivates female adolescents to seek SRH services. This finding is in conformity with a study conducted in Addis Ababa [55]. This finding highlights the importance of gender-sensitive approaches in the provision of ASRH services. Policymakers and practitioners should work towards developing interventions that are sensitive to gender differences, and aim to increase access to SRH services for all adolescents, regardless of gender.

Two-thirds of Adolescents had never talked to their parents about ASRH issues. Similar study findings were reported from a systematic review, where the pooled prevalence of adolescent-parent communications in Ethiopia is found to be only 43% [56]. However, adolescents who had discussions on ASRH issues with their parents were 4.5 times more likely to use the services [AOR = 4.53(2.52–8.16)] compared to those who had not. This finding is in agreement with other studies conducted in Harrar, Jimma, and Machakel. [32, 51, 56–58]. The underlying reason might be that having discussions on ASRH issues may build adolescents’ confidence and help them to expose their ASRH health concerns, understand consequences of SRH issues, and motivate them to use adequate services. The reasons that hindered communication between parents and adolescents in this study were that in the community it is deemed rude/disrespectful/taboo/stigma-associated behavior to discuss sexual topics and issues with one’s elders. This complements the systematic review findings, where adolescent-parent communication was low due to cultural taboo, shame, and lack of communication skills [57]. A national demographic survey report (EDHS) and qualitative findings from research among pastoralist communities in Bench Maji, reported restrictive sociocultural norms that prevent disclosure of information about sexual activity to unmarried adolescents [26, 30]. There, adolescents who discuss with peers were more likely to know and use ASRH services than those who talk with their parents. The possible explanation for this would be that there is more freedom to communicate about ASHR issues with peers/friends than with biological families. This finding highlights the need for further research on the relationship between communication about ASRH issues and the use of SRH services among adolescents. Future studies should aim to identify effective strategies to promote open and honest communication about ASRH issues between adolescents and their parents, peers and health care providers.

Adolescents, who had a risk perception for HIV/AIDS and unwanted pregnancies, had significant positive associations with ASRH service utilization. This result is confirmed by the studies conducted in Harrar [52] and Jimma [58]. The possible description may be that having a high level of risk perception might push adolescents to know their HIV status and seek Voluntary Counseling and Testing (VCT) services, which increases their ASRH service utilization. This finding highlights that risk perception is associated with increased use of ASRH services and shows the importance of developing interventions that promote risk perception among adolescents and provide them with the resources they need to protect their SRH.

A higher level of education was an enabling, supportive factor for ASRH service attendance among pastoralist adolescents – a finding which was supported by a systematic review discussing many other studies in Ethiopia [51, 52, 55]. Education is an essential enabling factor that improves SRH understanding because of relatively more disclosure of ASRH information in higher education institutions and associated behavioral change in adolescents.

Pastoralist adolescents’ religious and cultural devotion remains reasonably high, knowledge on alternative SRH service use (e.g. natural-contraceptive methods for family planning) is equally important to help religious pastoralist adolescents to make an informed decision in accordance with their faith. A similar finding was reported by Tigabu and colleagues, where Muslims were 65% less likely to utilize modern contraceptive services as compared to Orthodox Christians [29]. Adolescents and youth in this area live hold strong cultural values and beliefs. Furthermore, the offered services are insensitive to meet the unique requirements of pastoralist adolescents. Moving from place to place for a living appears to be depriving these communities of essential health services. Access and proper use of ASRH services have been hampered by remote locations with long distances to health services, high levels of illiteracy in the community, and harmful traditional beliefs [19, 32]. It is well known that these beliefs lead to poor SRH outcomes, such as FGC/M, early marriage, unprotected sex, and unplanned pregnancy [28, 30, 31, 44, 59]. Under the *absuma*^1^ marriage custom, Afar pastoralist girls typically marry a maternal cousin chosen by their parents in mid-adolescence (13–15 years). The girls and boys have no say in whom they marry or when [19, 60]. The finding implies that religious, social and cultural devotion among pastoral Adolescents remains high underlines the importance of promoting the use of alternative ASRH services and developing targeted interventions that take into account the specific needs and characteristics of different religious and cultural groups.

## Conclusion

The ASRH needs and challenges in Afar pastoralist areas are of critical concern. Despite the increased emphasis on the well-being and SRH of adolescents in Ethiopia, many adolescents in the pastoralist community do not have access to receive the recommended SRH services. Pastoralist adolescent populations have unique unmet SRH needs as evidenced by only one-fourth of the adolescents who have received SRH services at least once in the previous 12 months.

Adolescents having accessed SRH services once in the last 12 months. This low SRH uptake is primarily due to their pastoralist way of life, unavailability of services, lack of knowledge, societal stigma attached to accessing services, and the negative judgmental behavior from religious and cultural views in their families and the community. This study’s findings highlights a need for culturally sensitive adolescent-friendly SRH services in Afar, Ethiopia. This study’s findings have significant implications for policy and practice, particularly in improving SRH service use among Adolescents in the Afar region.

### Recommendations

Intensified efforts are required to improve the utilization of ASRH services among pastoralist adolescents in the Afar region. A regular health promotion strategy should be in place to reach all parts of the adolescent population. ASRH services should be easily accessible, acceptable, and affordable to pastoralist adolescents. The services should address and provide options to meet the real needs of pastoralist adolescents. This may include facilitated access to health care staff and sites for example at market and ceremony places, after-school, in the evening and/or during the weekend. Additionally, continued assessments, surveys, and consultations with adolescent youth advisory councils about the preferred time and delivery modalities in these ASRH facilities are essential.

Establishing and strengthening adolescent reproductive health clubs in and out of school might play a great role in understanding, awareness, and especially behavioral change leading to an increase in ASRH service utilization. Age-appropriate puberty education courses, starting with younger children as young as 9, need to be offered in formal schools and other informal community settings, as well as courses for parents to help them address children’s questions and concerns.

Afar regional health bureaus and Mille district health offices need to gradually shift from the existing practices to more effective “Gender-culture-context-appropriate” interventions so that the actual needs of adolescents are addressed in context. Trying to achieve improved long-term outcomes requires interaction with adolescents and adapting interventions to their needs, which at this vulnerable time of life are dynamic, confidential, and flexible by nature.

Further research should include the specific characteristics of pastoralist communities such as distance to health facilities, access to health extension services and food security variables in the analysis. In addition, recent literature suggests that the ongoing Afar-Issa conflict in the Afar region and the war in northern Ethiopia (Tigray) have significant implications for ASRH service use. It is therefore crucial to consider the impact of the war when developing strategies and interventions to improve the uptake of SRH services by AYs in the region.

### Strengths and limitations

One of the strengths of this study is the community-based approach among hard-to-reach pastoralist adolescents in rural Ethiopia, with an adequate sample size to detect the true status and factors. Information in pastoralist settings is often shared through spoken word (daxgu in Afar’Af) rather than through written records. This might make it difficult to give an accurate response on ASRH service use over time. Additionally, cultural norms around discussing sensitive topics like sex and reproductive health might have created difficulties in obtaining honest and accurate responses particularly from women and adolescents. However, anonymity and confidentiality were emphasized and the interviewers were of the same gender. Because the data collection was conducted retrospectively, recall bias and social desirability bias may have led to under- or over-reporting of the key findings. Finally, the study was conducted with pastoralist adolescents, this means that the results may not be generalizable to the entire Ethiopian youth population, which is socioeconomically, linguistically, and ethnically diverse. Researchers should consider these limitations when interpreting the study’s findings and future research should address these limitations to further advance our understanding of SRH service use among pastoralist AYs.

## Data Availability

The raw data are available from the corresponding author upon reasonable request.

## Footnotes

2 A community or village in Afar; smallest administrative unit in Ethiopia.

1 A traditional clan-based marriage system in Afar between maternal cousins.

## Abbreviations

ASRH: Adolescent Sexual Reproductive Health
AYS: Adolescents youths
DALYs: Disability-adjusted life years is a measure of overall disease burden, expressed as number of years lost due to ill-health, disability, or early death.
EDHS: Ethiopia Demographic Health Survey
SPSS: Statistical Package for the Social Sciences
FGC/M: female genital cutting/mutilation
STI: Sexually Transmitted Infections, VCT:Voluntarily Counselling and Testing
WHO: World Health Organization.
